# Social Isolation and Incident Post-Stroke Depression: A Population-Based Cohort Study

**DOI:** 10.1192/j.eurpsy.2026.10599

**Published:** 2026-07-31

**Authors:** Y. Ou, Y. Chen, M. Li, Y. Y. Liang

**Affiliations:** 1 Institute of Psycho-neuroscience, The Affiliated Brain Hospital, Guangzhou Medical University, Guangzhou, China; 2 Department of Psychology, The University of British Columbia, Vancouver, Canada

## Abstract

**Introduction:**

Post-stroke depression, a prevalent and persistent neuropsychiatric complication, adversely affects stroke recovery outcomes. Almost half of stroke survivors experience social isolation. Yet the contribution of social isolation to post- stroke depression development remains unclear.

**Objectives:**

We investigate the association between social isolation and incident depression in stroke survivors.

**Methods:**

We included 7,267 UK Biobank participants with prior stroke (mean±SD age =60.5 ±6.9 years, 55.6% male) without baseline depression. Degree of social isolation was assessed with three questions: 1) “Including yourself, how many people are living together in your household? (Include those who usually live in the house such as students living away from home during term, partners in the armed forces, or professions such as pilots)” (1 point for living alone); 2) “How often do you visit friends or family or have them visit you?” (1 point for answering rarely, never, or monthly); and 3) “Which of the following [leisure/social activities] do you engage in once a week or more often? You may select more than one” (1 point for none of the above). Incident post-stroke depression was ascertained from hospital records and death registries. We used Cox cause-specific hazards models to estimate hazard ratios (HRs) adjusted for demographics, socioeconomic status, comorbidities, health behaviours and physiological covariates. We also performed a relative importance analysis to rank risk factors. We calculated the Percentage of Excess Risk Mediated (PERM) by grouping explanatory covariates into categories, such as medication, behaviors, and comorbidities.

**Results:**

Over a median 10.2-year follow-up, 591 stroke survivors developed depression. Compared with the least isolated participants, the most isolated group showed a significantly higher risk of depression (HR: 1.35, 95% CI: 1.07-1.70). In a risk-factor ranking, social isolation ranks higher than traditional risk factors, including hypertension and obesity, as a predictor of post-stroke depression. Health behaviors mediated the association between social isolation and elevated depression risk most strongly, accounting for 32-44% (PERM) of the effect. This mediating effect was significantly greater than that explained by medication, comorbidities, and other covariates.

**Conclusions:**

In this population-based cohort study of UK Biobank participants with stroke, social isolation was significantly associated with an increased risk of post-stroke depression. These findings suggest that interventions targeting social connectivity may improve clinical outcomes in stroke survivors.

**Disclosure of Interest:**

None Declared

